# Hydrangenol inhibits the proliferation, migration, and invasion of EJ bladder cancer cells via p21^WAF1^-mediated G1-phase cell cycle arrest, p38 MAPK activation, and reduction in Sp-1-induced MMP-9 expression

**DOI:** 10.17179/excli2018-1361

**Published:** 2018-06-06

**Authors:** Seung-Shick Shin, Myeong-Cheol Ko, Yu-Jin Park, Byungdoo Hwang, Sung Lyea Park, Wun-Jae Kim, Sung-Kwon Moon

**Affiliations:** 1 Department of Food Science and Nutrition, Jeju National University, Jeju 63243, South Korea; 2 Department of ICT Convergence Engineering, College of Science and Technology, Konkuk University, Chungju, Chungbuk 27478, South Korea; 3 Department of Food and Nutrition, Chung-Ang University, Anseong, Kyung-gi 17546, South Korea; 4 Department of Urology, Chungbuk National University, Cheongju, Chungbuk 28644, South Korea

**Keywords:** hydrangenol, proliferation, invasion, migration, MMP-9

## Abstract

Hydrangenol is a dihydroisocoumarin that is mainly obtained from *Hydrangea macrophylla*. Recently, hydrangenol has garnered attention since several studies have reported that it has anti-inflammatory, anti-allergic, anti-diabetic, and anti-malarial activities. However, there have been few studies on the effect of hydrangenol on oncogenesis. In this study, we evaluated the anti-cancer activity of hydrangenol against the EJ bladder cancer cell line. Hydrangenol significantly inhibited the proliferation of EJ cells in a dose-dependent manner with an IC_50_ of 100 µM. Flow cytometry and immunoblotting experiments indicated that EJ cells were arrested in the G1-phase of the cell cycle and showed reduced expression of CDK2, CDK4, cyclin D1, and cyclin E mediated via the upregulation of p21^WAF1^. Hydrangenol increased the phosphorylation of p38 MAPK without affecting the phosphorylation of ERK and JNK. In addition, hydrangenol significantly inhibited the migratory and invasive activities of EJ cells by suppressing the enzymatic activity of MMP-9. Electrophoretic mobility shift assays suggested that the inhibition of MMP-9 activity by hydrangenol was attributable to its suppression of the Sp-1 transcription factor binding activity. This study is the first report on the mode of action of hydrangenol as an inhibitor of bladder cancer. We believe that these results provide novel insights that could aid the development of hydrangenol-based chemotherapeutic agents.

## Introduction

Bladder cancer is one of the most common cancers worldwide and the fourth most common cancer in men within the United States of America. The fatality of bladder cancer at advanced stages is remarkably high, and according to annual statistics reported by the American Cancer Society, the incidence of urinary bladder cancer has been predicted to be 81,190 new cases and 17,240 deaths in 2018 (Siegel et al., 2018[27]). Bladder cancer occurs in the inner lining of the bladder and typically progresses to transitional cell carcinoma. During the neoplastic progression, this transitional cell carcinoma acquires the capabilities of inter-tissue migration and invasion (Park et al., 2014[25]), which may lead to fatality for patients with bladder cancer. Therefore, understanding the molecular events associated with the uncontrolled proliferation of bladder cancer cells is a critical step in the development of novel therapeutic reagents for the management of this life-threatening disease. 

Malignantly transformed cells react to treatment with chemotherapeutic reagents through the modulation of key regulators of early responsive kinases including mitogen-activated protein kinases (MAPKs) and PI3K/AKT (Gerhardt et al., 2014[9]; Zheng et al., 2014[34]). A cascade of molecular signals originating from these kinases eventually reaches the nucleus of the cell and modulates the activities of cell cycle regulators including cyclin-dependent kinases (CDKs), cell cycle inhibitors (CKIs), and cyclins. In eukaryotes, the cell cycle is divided into four phases, comprising two gap phases (G1 and G2), S phase, and M phase (Lee and Yang, 2001[14]; Li and Blow, 2001[15]). In recent decades, protein regulators of the G1 cell cycle phase have drawn attention as promising molecular targets of certain cancer drugs such as palbociclib (Michel et al., 2016[20]) and the experimental agents UNC-01 (Li et al., 2012[17]) and E7070 (Haddad et al., 2004[10]). At the end of the G1 phase, the G1 checkpoint regulates the progression from G1 to S phase, which is characterized by the formation of complexes between cyclins and CDKs (Sherr, 1996[26]). CDKs are negatively regulated by some chemotherapeutic drugs, which results in the upregulation of the CKIs p21^WAF1^ and p27^KIP1^ (Chen et al., 2016[5]; Izutani et al., 2012[11]). In bladder cancer, the expression of matrix metalloproteinases (MMPs), which are extracellular endopeptidases, has been reported to be associated with the capacities of cancer cells for migration and invasion. Thus, MMPs have been suggested as a promising target of chemotherapeutic drugs (Bianco et al., 1998[2]; Davies et al., 1993[8]; Nutt et al., 2003[22]; Vasala et al., 2008[30]). In particular, Davies and coworkers reported that the expression of MMP-9 was significantly higher in the tumor tissues of bladder cancer patients as compared to non-cancerous tissues from the same patients. They also showed that the expression of MMP-2 and MMP-9 increased as the tumor grade increased (Davies et al., 1993[8]). In addition, Bianco and colleagues reported that MMP-9 expression is closely correlated with the onset and progression of bladder cancer (Bianco et al., 1998[2]).

Hydrangenol is a naturally occurring dihydroisocoumarin that is mainly found in *Hydrangea* *macrophylla*. Recently, hydrangenol has drawn attention since it has been reported to exhibit anti-inflammatory (Kim et al., 2016[13]), anti-microbial (Nozawa et al., 1981[21]), anti-diabetic (Zhang et al., 2007[33]), anti-allergic (Matsuda et al., 1999[19]), and anti-malarial (Kamei et al., 2000[12]) activities. Kim and colleagues reported that hydrangenol exhibited a strong anti-allergic activity by suppressing NF-κB signaling and activating the Nrf2-mediated HO-1 pathway in lipopolysaccharide-stimulated macrophages (Kim et al., 2016[13]). Sugie and colleagues (1998[28]) reported that hydrangenol showed a tendency to decrease the onset and progression of tumors in the entire intestine induced by azoxymethane; however they concluded that the effect was not statistically significant (Sugie et al., 1998[28]). Despite these reports, few studies have investigated the potential anti-cancer activity of hydrangenol. In the present study, we investigated whether hydrangenol shows an anti-cancer activity against human bladder cancer cells and identified key regulators involved in its mode of action. We believe that the results provide a better understanding of hydrangenol and indicate that it may represent a candidate chemotherapeutic agent for bladder cancer.

## Materials and Methods

### Materials

Hydrangenol was purchased from Coresciences Co. (Seoul, South Korea). Antibodies against CDK2, CDK4, cyclin D1, cyclin E, p21^WAF1^, p27^KIP1^, p53, and GAPDH were purchased from Santa Cruz Biotechnology (Dallas, TX, USA). Total ERK, p38, JNK, AKT, phospho-ERK, phospho-p38, phospho-JNK, and phospho-AKT antibodies were purchased from Cell Signaling Technology (Danvers, MA, USA). The polyclonal MMP-9 antibody was purchased from Chemicon (Temecula, CA, USA). The nuclear extract kit and the electrophoretic mobility shift assay (EMSA) gel shift kit were obtained from Panomics (Fremont, CA, USA). The p38-specific inhibitor SB203585 was purchased from Calbiochem (San Diego, CA, USA). 

### Cell culture 

The human bladder cancer EJ cells were kindly provided by Dr. Wun-Jae Kim (Department of Urology, Chungbuk National University, Chungbuk, South Korea). EJ cells were grown in Dulbecco's modified Eagle's medium supplemented with 10 % fetal bovine serum (FBS), 100 U/mL penicillin, and 100 μg/mL streptomycin at 37 °C in a 5 % CO_2_ humidified incubator. 

### Hydrangenol treatment, cell viability assay, and cell counting

Cell viability was determined by the 3-(4,5-dimethylthiazol-2-yl)-2,5-diphenyltetrazolium bromide (MTT) assay. Briefly, 5 × 10^3^ cells per well were plated in 96-well plates and incubated with triacanthine (0, 100, 200, 400, and 600 µM) for 24 h. Then, the medium was removed, and fresh medium containing 10 μL of 5 mg/mL MTT was added. After 1 h, the medium was removed and replaced with 100 μL of dimethyl sulfoxide. The absorbance at 540 nm was measured using a fluorescent plate reader. The morphology of the cell was photographed using a phase-contrast microscope. For the counting of viable cells, EJ cells (3 × 10^5^ cells/well) were plated in 6-well plates and allowed to settle on the surface of the plates for 2 h. Then, the cells were treated with hydrangenol at different concentrations (0, 50, 100, and 200 µM) for 24 h. The cells were detached with 0.25 % trypsin containing 0.2 % ethylenediaminetetraacetic acid (EDTA) (Thermo Fisher Scientific, Waltham, MA, USA). The detached cells were mixed with 50 μL of 0.4 % trypan blue (Sigma-Aldrich, St. Louis, MO, USA) by gentle pipetting. Cells were counted using a hemocytometer.

### Flow cytometry (cell cycle) analysis

EJ cells were trypsinized and fixed with 5 mL of 70 % ethanol. After washing once with 1× ice-cold phosphate buffered saline (PBS), the cells were harvested by centrifugation. Then, the cells were treated with RNase (1 mg/mL) followed by propidium iodide (50 mg/mL). The proportion of cells in each cell-cycle phase was measured using a flow cytometer (FACStar™, BD Biosciences, San Jose, CA) equipped with the BD Cell Fit software. 

### Immunoblotting and immunoprecipitation

Cells were washed twice with 1× cold PBS. Then, the cells were lysed in 200 μL of a lysis buffer containing 50 mM 4-(2-hydroxyethyl)-1-piperazineethanesulfonic acid (HEPES) (pH 7.5), 150 mM NaCl, 1 mM EDTA, 1 mM dithiothreitol (DTT), 2.5 mM egtazic acid (EGTA), 10 mM β-glycerophosphate, 0.1 mM Na_3_VO_4_, 1 mM NaF, 1 mM phenylmethylsulfonyl fluoride (PMSF), 10 % glycerol, 0.1 % Tween^®^ 20, 10 μg/mL leupeptin, and 2 μg/mL aprotinin. The cells were scraped and transferred into 1.5 mL tubes. The lysates were incubated on ice for 10 min and then centrifuged at 10,000 × *g* for 10 min at 4 °C. The amount of protein in each whole cell lysate was measured by a bicinchoninic acid protein assay reagent kit (Thermo Fisher Scientific). Twenty-five micrograms of each protein lysate was loaded onto a 10 % polyacrylamide gel under denaturing conditions. The separated proteins were transferred onto nitrocellulose membranes (Hybond^®^, GE Healthcare Life Sciences, Marlborough, MA, USA). The membranes were blocked in 5 % skim milk, followed by incubation with primary antibodies overnight. Next day, the membranes were incubated with a peroxidase-conjugated secondary antibody for 90 min. The immunocomplexes were analyzed using a chemiluminescence reagent kit (GE Healthcare Life Sciences). For immunoprecipitation assays, equal amounts of cell lysates were incubated with the indicated antibodies overnight at 4 °C. Then, protein A-Sepharose^®^ beads (Santa Cruz Biotechnology) were added to the immunocomplexes and incubated at 4 °C for 2 h. The immunoprecipitated protein complexes were washed with 1 × lysis buffer three times, followed by incubation in sodium dodecyl sulfate polyacrylamide gel electrophoresis (SDS-PAGE) sample buffer containing β-mercaptoethanol (Bio-Rad Laboratories, Richmond, CA, USA). Then, the protein complexes were separated by SDS-PAGE. Experiments were repeated at least three times.

### Wound-healing migration assay

EJ cells were grown and seeded in 6-well plates (3 × 10^5^ /well). To exclude proliferation-mediated migration, cells were pre-incubated with 5 μg/mL mitomycin C (Sigma-Aldrich) for 2 h. Assigned areas of the cell surface were scratched with a 2-mm-wide pipette tip. After washing with 1× PBS three times, the cells were incubated with culture medium in the presence or absence of hydrangenol (0, 50, 100, and 200 µM) for 24 h. The migration of the cells into the scratched area was evaluated by measuring the remaining size of the scratch wound with comparison to the control without hydrangenol treatment. Morphology changes of the cells that were induced by hydrangenol treatment were photographed using an inverted microscope at 40× magnification. 

### Boyden chamber invasion assay

The invasive potential of hydrangenol-treated EJ cells was measured using Matrigel^®^-coated 6.5 mm transwell plates with 8 μm pores (Sigma-Aldrich). Briefly, 2.5 × 10^4^ cells were pre-incubated in serum-free medium containing mitomycin C (5 μg/mL) for 2 h. Then, the cells were plated in the upper chamber. Culture medium containing 10 % FBS as an attractant was added to the lower chamber. After 24 h, cells that had migrated to the lower chamber were stained and photographed. 

### Zymography

Cells were treated with different concentrations of hydrangenol (0, 50, 100, and 200 µM) in a medium containing FBS for 24 h. Then, the culture medium was changed to an FBS-free conditioned medium for an additional 24 h. Next, the cultured conditioned medium was collected and electrophoresed using a polyacrylamide gel containing 0.25 % gelatin. The gel was washed twice with 2.5 % Triton X-100™ for 15 min at room temperature. Then, the gel was incubated in a buffer containing 50 mM Tris-HCl, 150 mM NaCl, and 10 mM CaCl_2_, pH 7.5 at 37 °C overnight. The gel was stained with 0.2 % Coomassie blue, destained with a destaining solution (10 % acetic acid and 10 % methanol in distilled water), and photographed on a light box. Gelatinase activity was visualized as a white zone in a dark blue field.

### Nuclear extracts and EMSA

EJ cells were treated with hydrangenol (0, 100, and 200 µM) for 24 h. Nuclear extracts were prepared with a nuclear extraction kit (Panomics). Briefly, EJ cells were collected by centrifugation, washed, and resuspended in a buffer containing 10 mM HEPES (pH 7.9), 10 mM KCl, 1 mM DTT, 0.5 mM PMSF, 0.1 mM EDTA, and 0.1 mM EGTA. After incubation on ice for 15 min, the cells were lysed with 0.5 % NP-40. The nuclear pellet was harvested by centrifugation, followed by extraction in an ice-cold high-salt buffer [20 mM HEPES (pH 7.9), 400 mM NaCl, 1 mM PMSF, 1 mM DTT, 1 mM EDTA, and 1 mM EGTA] at 4 °C for 15 min. After centrifugation, the supernatant containing the nuclear extract was obtained. The concentration of total protein was measured using a bicinchoninic acid protein assay reagent kit (Thermo Fisher Scientific). Twenty micrograms of the nuclear extract were preincubated at 4 °C for 30 min with a 100-fold excess of an unlabeled oligonucleotide spanning the −79 position of the *MMP9* cis-acting element. The oligonucleotide sequences were as follows: AP-1, CTGACCCCTGAGTCAGCACTT; NF-κB, CAGTGGAATTCCCCAGCC; and Sp-1, GCCCATTCCTTCCGCCCCCAGATGAA-GCAG. Then, the reaction mixture was incubated in a buffer [25 mM HEPES (pH 7.9), 50 mM NaCl, 0.5 mM DTT, 0.5 mM EDTA, and 2.5 % glycerol] at 4 °C for 20 min with 2 μg of poly dI/dC and 5 fmol (2 × 10^4^ cpm) of a Klenow end-labeled (^32^P ATP) 30-mer oligonucleotide spanning the DNA-binding site of the *MMP9* promoter. The reaction mixture was analyzed by electrophoresis using a 6 % polyacrylamide gel. Then, the gel was exposed to X-ray film overnight. The gray values of the blots were measured using the ImagePro Plus 6.0 software (Media Cybernetics, Rockville, MD, USA).

### Statistical analysis

Where appropriate, data are presented as the mean ± standard deviation. Data were evaluated by factorial analysis of variance and Fisher's least significant difference test where appropriate. Statistical significance was considered at *P* < 0.05.

## Results

### Hydrangenol inhibits the proliferation of EJ bladder cancer cells in a dose-dependent manner

**Figure 1:**
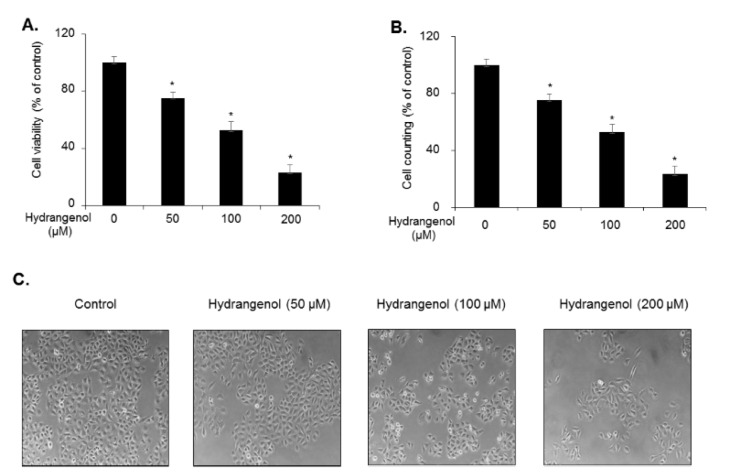
Inhibition of the proliferation of EJ bladder cancer cells by hydrangenol. (A) EJ cells were treated with different concentrations of hydrangenol for 24 h, followed by the MTT proliferation assay. (B) The cells were stained with trypan blue and their viability was evaluated by counting viable cells. (C) Changes in the cellular morphology of hydrangenol-treated EJ bladder carcinoma cells. Values in the bar graphs represent the mean ± standard deviation (SD) of three independent experiments; *P < 0.05, compared with the control group.

To investigate the potential of hydrangenol as a therapeutic reagent for bladder cancer, we examined its inhibitory activity against the proliferation of EJ cells. Cells were incubated with different concentrations (0, 50, 100, and 200 µM) of hydrangenol for 24 h and cellular viability was evaluated using the MTT assay (Figure 1A(Fig. 1)). As shown in Figure 1A(Fig. 1), treatment with hydrangenol significantly inhibited the proliferation of EJ carcinoma cells in a dose-dependent manner with an IC_50_ of approximately 100 µM. The number of viable cells was also measured by trypan blue staining, which exhibited a similar result to the MTT assay (Figure 1B(Fig. 1)). Treatment with hydrangenol dose-dependently resulted in an increased proportion of EJ cells with a rounded morphology, which may indicate heightened cellular stress (Figure 1C(Fig. 1)).

### Hydrangenol leads to G1-phase cell cycle arrest in EJ cells

**Figure 2:**
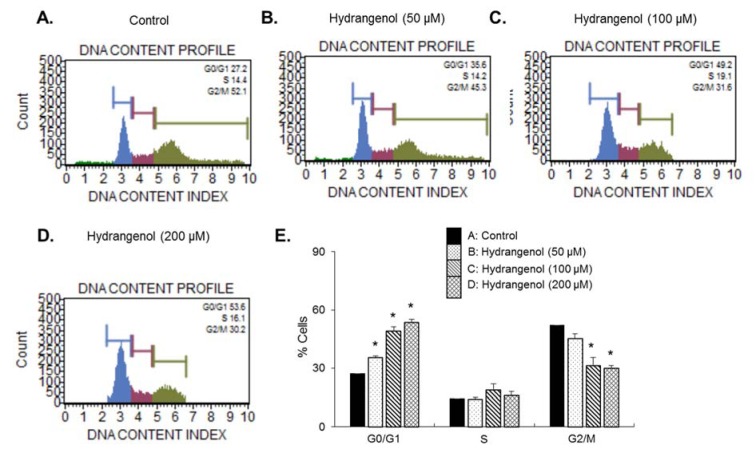
Cell cycle distribution of EJ cells treated with hydrangenol. Hydrangenol led to G1 cell cycle phase arrest in EJ cells. The cells were treated with hydrangenol at concentrations of 0 (A), 50 (B), 100 (C), and 200 (D) μM. Flow cytometric analysis was performed to determine the cell cycle phase distribution in EJ cells treated with hydrangenol. (E) The percentage of cells in each phase is presented. Values in the bar graph represent the mean ± SD of three independent experiments.

To better understand the mode of action of hydrangenol, we examined the cell cycle distribution of hydrangenol-treated EJ bladder cancer cells. The cells were treated with different concentrations of hydrangenol (0, 50, 100, and 200 µM) for 24 h, followed by flow cytometry analysis. The results showed that a significant proportion of hydrangenol-treated EJ cells were arrested in the G1 cell cycle phase as compared with the vehicle-treated control cells. The proportion of cells arrested in the G1 phase showed a dose-dependent relationship with the concentration of hydrangenol (Figure 2(Fig. 2)). These data suggest that hydrangenol inhibits the proliferation of EJ bladder carcinoma cells through the induction of G1 cell cycle phase arrest. 

### Hydrangenol downregulates cyclin/CDK complex formation via the upregulation of p21^WAF1^

**Figure 3:**
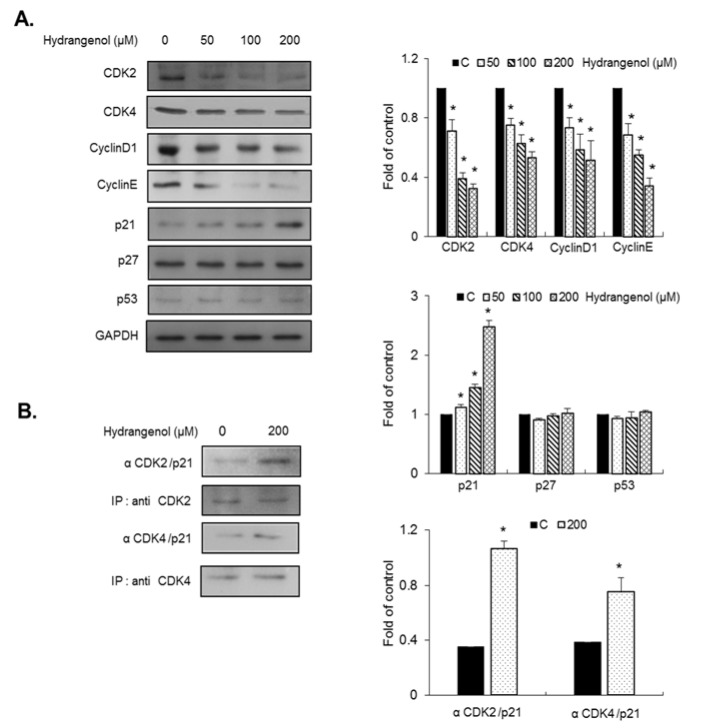
Immunoblots and immunoprecipitations of cell cycle regulators in hydrangenol-treated EJ cells. EJ cells were treated with hydrangenol at the indicated concentrations for 24 h. Protein lysates were prepared and analyzed by sodium dodecyl sulfate polyacrylamide gel electrophoresis. (A) Changes in the protein abundance of CDK2, CDK4, cyclin D1, cyclin E, p21^WAF1^, p27^KIP1^, and p53 were measured by immunoblotting. GAPDH was used as a loading control. (B) After treatment with hydrangenol at 0 and 200 μM, EJ cell lysates were prepared and immunoprecipitation was performed with specific antibodies against CDK2 and CDK4 followed by immunoblotting with a p21^WAF1^ antibody. Bar graphs show the relative strength of each protein band represented as a fold change compared to the control. Each value was presented as the mean ± SD of three independent experiments; *P < 0.05, compared with the control group.

To better understand the molecular mechanism of hydrangenol, we examined the abundance of key proteins that regulate the progression of the cell cycle from the G1 phase to the S phase. EJ bladder cancer cells were incubated with different concentrations of hydrangenol (0, 50, 100, and 200 µM) for 24 h. Protein lysates from the hydrangenol-treated EJ cells were prepared and subjected to immunoblot analyses. As exhibited in Figure 3A(Fig. 3), the abundances of CDK2, CDK4, cyclin D1, and cyclin E significantly decreased with hydrangenol treatment in a dose-dependent manner (Figure 3A(Fig. 3)). Next, we examined whether hydrangenol modulates the abundances of cell cycle inhibitors including p21^WAF1^, p27^KIP1^, and p53, since many chemotherapeutic reagents lead to the upregulation of these inhibitors (Wang et al., 2013[31]; Yuan et al., 2015[32]). The abundance of p21^WAF1^ was significantly and dose-dependently upregulated by hydrangenol treatment. However, the abundances of p27^KIP1^ and p53 were unchanged by hydrangenol treatment (Figure 3A(Fig. 3)). To confirm the immunoblotting results, we performed immunoprecipitation experiments with anti-CDK2 and anti-CDK4 antibodies, followed by immunoblotting with an anti-p21^WAF1^ antibody (Figure 3B(Fig. 3)). The results showed that hydrangenol promoted the binding of p21^WAF1^ to both CDK2 and CDK4, which resulted in the suppression of the G1-to-S cell cycle phase transition. Taken together, these results clearly indicate that hydrangenol suppresses the proliferation of EJ bladder carcinoma cells by inhibiting the formation of complexes between CDKs and cyclins via a p21^WAF1^-dependent pathway.

### Hydrangenol activates p38 MAPK, but not ERK and JNK, in EJ bladder cancer cells

**Figure 4:**
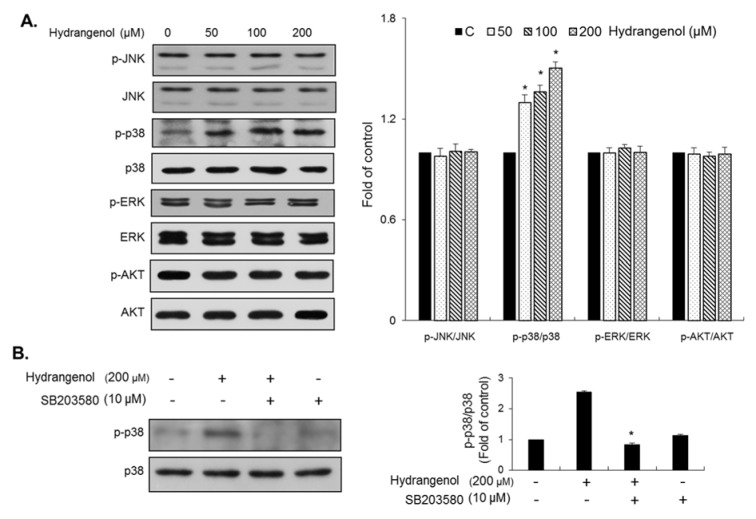
Changes in the phosphorylation of MAPKs and AKT in hydrangenol-treated EJ cells. EJ cells were treated with different concentrations (0, 50, 100, and 200 μM) of hydrangenol and protein lysates were prepared. (A) Immunoblotting was performed with specific antibodies against phospho-ERK, ERK, phospho-p38, p38, phospho-JNK, JNK, AKT, and phospho-AKT. The ratio of the phosphorylated to the unphosphorylated form was measured and presented as a fold change compared to the control. Each value was presented as the mean ± SD of three independent experiments; *P < 0.05, compared with the control group. (B) EJ cells were preincubated with or without SB203580, which is a specific inhibitor of p38, followed by incubation in the presence or absence of hydrangenol. Each value was presented as the mean ± SD of three independent experiments; *P < 0.05, compared with the treatment of hydrangenol only.

Several lines of evidence have suggested that early responsive regulators such as the MAPKs (ERK, JNK, and p38) and PI3K-AKT participate in the progression of bladder cancer (Dangle et al., 2009[6]; Gerhardt et al., 2014[9]). Thus, we examined whether the abundances of these proteins would be altered by treatment with hydrangenol. As shown in Figure 4A(Fig. 4), p38 MAPK activation was significantly increased by hydrangenol treatment in a dose-dependent manner in EJ cancer cells. Upon treatment with 200 µM hydrangenol, phospho-p38 MAPK was increased by up to 1.5-fold as compared to the vehicle-treated control. However, the activation of ERK and JNK was unchanged by hydrangenol treatment (Figure 4A(Fig. 4)). Immunoblotting results also showed that the mode of action of hydrangenol did not involve AKT activation (Figure 4A(Fig. 4)). To confirm the association of p38 MAPK with the molecular effect of hydrangenol in EJ cancer cells, we pre-incubated the EJ cells with a specific inhibitor of p38 MAPK, SB203580, followed by treatment with 200 µM hydrangenol. As shown in Figure 4B(Fig. 4), the pre-treatment of EJ cells with SB203580 eliminated the activation of p38 MAPK, which clearly suggests that the activation of p38 MAPK was attributed to the hydrangenol treatment. Taken together, these results indicate that p38 MAPK activation was involved in the hydrangenol-mediated inhibition of the proliferation of EJ bladder carcinoma cells.

### Hydrangenol reduces the migratory and invasive capacities of EJ cancer cells

**Figure 5:**
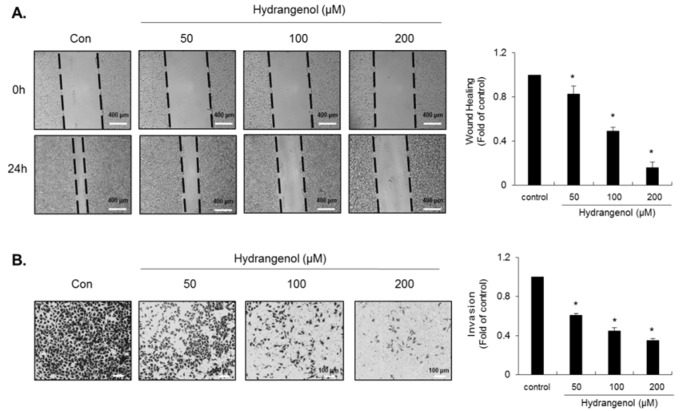
Inhibition of the migratory and invasive activities of EJ cells by hydrangenol. (A) Treatment with hydrangenol inhibited EJ cell migration. The cells were grown for 24 h and the cell monolayer was scratched with a pipette tip. The cells were then incubated with hydrangenol (0, 50, 100, and 200 μM) for 24 h. The distance of cell migration was measured by photographs using an inverted microscope at 40× magnification. (B) An invasion assay was performed using Matrigel^®^-coated transwell chambers. EJ cells were plated onto the upper chamber and incubated with hydrangenol for 24 h. Cells invading the lower surface of the membrane were stained with crystal violet and visualized using a light microscope. The morphology of the cells was observed with a phase-contrast inverted microscope. The bar graphs show the number of invading cells represented as a fold change relative to the control. Values in the bar graphs represent the mean ± SD from three different experiments. *P < 0.05, compared to the control.

Transformed bladder cells often acquire the ability to invade other tissues through enhanced migratory and invasive capacities. We investigated whether hydrangenol treatment influences the migration and invasion of EJ cancer cells. To this end, we utilized *in vitro* wound-healing migration and transwell invasion assays. To distinguish cellular migration from proliferation-mediated movement, we pre-incubated EJ cells with mitomycin C (5 μg/mL) for 2 h and then incubated the cells in the presence or absence of hydrangenol for a further 24 h. As shown in Figure 5A(Fig. 5), the migration of EJ cells was significantly reduced by hydrangenol treatment in a dose-dependent manner. The migratory activity of EJ cells was reduced by approximately 85 % by treatment with 200 µM hydrangenol as compared to the control (Figure 5A(Fig. 5)). We also investigated whether hydrangenol affects the invasiveness of EJ cancer cells using Matrigel^®^-coated transwell plates. As shown in Figure 5B(Fig. 5), the invasiveness of EJ cells was significantly reduced by hydrangenol treatment in a dose-dependent manner. The number of cells invading through the transwell membrane was reduced by approximately 65 % after treatment with 200 µM hydrangenol as compared to the control (Figure 5B(Fig. 5)). These data clearly indicate that hydrangenol significantly inhibits the migration and invasion of EJ bladder carcinoma cells.

### Hydrangenol inhibits the activity of MMP-9 by suppressing the binding activity of the Sp-1 transcription factor

**Figure 6:**
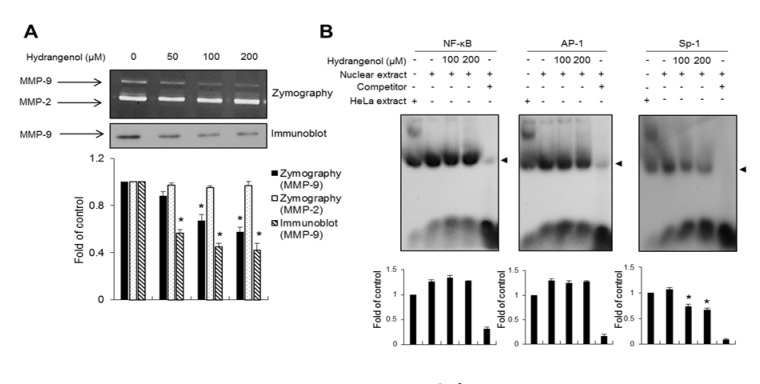
Hydrangenol-mediated inhibition of MMP-9 via suppression of the binding activity of Sp-1. (A) The matrix-degrading activities of MMP-2 and -9 were measured by gelatin zymography. The conditioned media of EJ cells in the presence of hydrangenol were analyzed by sodium dodecyl sulfate polyacrylamide gel electrophoresis under non-denaturing conditions. The lower panel shows the results of an immunoblot for MMP-9. The bar graph shows the activities of MMP-2 and -9 in hydrangenol-treated EJ cells relative to those of the control. The results are presented as the mean ± SD from three different experiments. *P < 0.05, compared to the control. (B) Nuclear extracts were collected from the cells treated with hydrangenol (0, 100, and 200 μM). The binding activities of NF-κB, AP-1, and Sp-1 were measured by an electrophoretic mobility shift assay.

The aberrant expression of proteinases is often observed in cancer cells. In bladder cancer, endopeptidases such as MMPs have been reported to be key determinants of metastatic progression (Bianco et al., 1998[2]; Dano et al., 1999[7]). Therefore, we investigated whether hydrangenol affects the activity of MMPs in EJ bladder cancer cells. We utilized gelatin zymography to evaluate changes in the enzymatic activities of MMP-2 and MMP-9 in hydrangenol-treated EJ cells. As exhibited in Figure 6A(Fig. 6), the proteolytic activity of MMP-9 was significantly inhibited by the hydrangenol treatment; however, the activity of MMP-2 was unchanged. The activity of MMP-9 was reduced by approximately 60 % in the cells treated with 200 µM hydrangenol as compared to the control cells (Figure 6A(Fig. 6)). To better understand the molecular mechanism underlying the hydrangenol-mediated inhibition of MMP-9 activity, we used the EMSA technique. To this end, we designed DNA fragments containing nucleotide sequences corresponding to the transcription factor-responsive elements of NF-κB, AP-1, and Sp-1, which are all present in the promoter region of *MMP9*. EJ cells were treated with 100 and 200 µM hydrangenol for 24 h, followed by EMSA. As shown in Figure 6B(Fig. 6), the *MMP9* promoter-binding activity of Sp-1 was significantly reduced by hydrangenol treatment; however, the binding activities of NF-κB and AP-1 were not altered (Figure 6B(Fig. 6)). Taken together, these results unequivocally suggest that hydrangenol inhibits the migration and invasiveness of EJ cancer cells by suppressing *MMP9* transcription through reducing the binding activity of Sp-1. 

## Discussion

For decades, solvent extracts of *H.* *macrophylla* have been used as a folk phytomedicine in eastern Asia to treat liver diseases, diabetes, and malaria (Akanda et al., 2017[1]; Kamei et al., 2000[12]). Although the diverse bioactivities of the extracts from *H. macrophylla* have drawn attention, the undefined nature of the extracts has limited their utilization as medicines. Hydrangenol is one of the bioactive compounds produced by plants of the Hydrangeaceae family. In this study, we investigated the molecular mechanisms underlying the anti-cancer activity of hydrangenol against bladder carcinoma cells. To our knowledge, this is the first study that has systematically investigated the mode of action of hydrangenol in carcinogenesis.

We first evaluated the anti-cancer efficacy of hydrangenol. Because few previous studies have investigated whether hydrangenol shows anti-cancer activities, the scope for making direct comparisons with the current results is limited. Sugie and colleagues (1998[28]) reported that juglone, plumbagin, and hydrangenol inhibited azoxymethane-induced intestinal carcinogenesis in a rat model. They reported that hydrangenol reduced the incidence and the multiplicity of azoxymethane-induced tumors; however, they concluded that the effect was not statistically significant. In our study, we observed that treatment with hydrangenol significantly inhibited the proliferation of EJ bladder carcinoma cells in a dose-dependent manner. Although the previous *in vivo* study cannot easily be compared with our *in vitro* study, such disparate results may be derived from tissue-specific effects or different origins of the malignant cells. We performed a flow cytometry analysis, which showed that the hydrangenol-treated EJ cells accumulated in the G1 phase of the cell cycle. This result was interesting because many chemotherapeutic drugs such as paclitaxel, tamoxifen, and 5-fluorouracil cause cancer cells to undergo growth arrest in the G1 cell cycle phase (Li et al., 2004[16]; Lin et al., 1998[18]; Osborne et al., 1983[23]). In mammalian cells, the cell cycle transition from G1 to S phase is mainly regulated by the CDK/cyclin complexes (Li and Blow, 2001[15]; Owa et al., 2001[24]). In our data, hydrangenol significantly reduced the protein abundances of CDK2 and CDK4 as well as their respective binding partners, cyclin E and cyclin D1. Among cell-cycle inhibitors, p21^WAF1^, but not p27^KIP1^ or p53, was shown to participate in the mode of action of hydrangenol. An immunoprecipitation assay using the p21^WAF1^ antibody showed that hydrangenol promotes the binding of p21^WAF1^ to CDK2 and CDK4, which results in the suppression of the G1-to-S cell cycle phase transition. Given that the abundance of p21^WAF1^ is modulated by hydrangenol, it may be utilized as a molecular marker to monitor the responses of patients undergoing chemotherapy with hydrangenol. Considering the mechanisms that regulate the proliferation of cancer cells, the key roles of the early responsive kinases, MAPKs (ERK, JNK, and p38 MAPK) and PI3K-AKTs have been well established (Chambard et al., 2007[3]; Chang et al., 2003[4]; Vadlakonda et al., 2013[29]). In our data, hydrangenol treatment induced the activation of p38 MAPK; however, ERK, JNK, and AKT did not participate in the mechanism of action of hydrangenol.

In addition, hydrangenol significantly inhibited the migratory and invasive activities of EJ cancer cells in a concentration-dependent manner. Using a gelatin zymography assay, we showed that the hydrangenol-mediated inhibition of invasiveness was associated with inhibition of the enzymatic activity of MMP-9. Because MMP-9 is one of the key factors by which bladder cancer cells acquire the capacity to metastasize and invade adjacent tissues or distant organs, the inhibitory activity of hydrangenol against MMP-9 might be particularly valuable. Using oligonucleotide sequences corresponding to the transcription factor-responsive elements of NF-κB, AP-1, and Sp-1, which are all found in the promoter region of *MMP9*, we found that inhibition of the transcription factor Sp-1 by hydrangenol was responsible for the reduction of MMP-9 activity.

In conclusion, these data suggest that hydrangenol has an anti-cancer activity as demonstrated by its inhibitory effects on the proliferation, migration, and invasion of EJ bladder carcinoma cells. Hydrangenol treatment led to growth arrest in the G1 cell cycle phase through the upregulation of p21^WAF1^, while p38 MAPK was the only kinase upregulated by hydrangenol treatment. The inhibition of the migratory and invasive activities of EJ cells was at least partly mediated by the suppression of the enzymatic activity of MMP-9 via inhibition of the binding activity of the transcription factor Sp-1. The data from this study provide information about hydrangenol that may support the development of chemotherapeutic reagents for bladder cancer patients.

## Notes

Seung-Shick Shin and Myeong-Cheol Ko contributed equally as first authors.

## Acknowledgement

This work was supported by the National Research Foundation of Korea (NRF) grant funded by the Korea government (MSIP) (No. 2017R1A2B4009384). The research was also supported by the Chung-Ang University Research Scholarship Grants in 2017. 

## Conflict of interest

The authors declare that they have no conflict of interest.

## References

[1] Akanda MR, Tae HJ, Kim IS, Ahn D, Tian W, Islam A (2017). Hepatoprotective role of Hydrangea macrophylla against sodium arsenite-induced mitochondrial-dependent oxidative stress via the inhibition of MAPK/ Caspase-3 pathways. Int J Mol Sci.

[2] Bianco FJ, Gervasi DC, Tiguert R, Grignon DJ, Pontes JE, Crissman JD (1998). Matrix metalloproteinase-9 expression in bladder washes from bladder cancer patients predicts pathological stage and grade. Clin Cancer Res.

[3] Chambard JC, Lefloch R, Pouyssegur J, Lenormand P (2007). ERK implication in cell cycle regulation. Biochim Biophys Acta.

[4] Chang F, Steelman LS, Shelton JG, Lee JT, Navolanic PM, Blalock WL (2003). Regulation of cell cycle progression and apoptosis by the Ras/Raf/MEK/ ERK pathway. Int J Oncol.

[5] Chen G, Gong R, Shi X, Yang D, Zhang G, Lu A (2016). Halofuginone and artemisinin synergistically arrest cancer cells at the G1/G0 phase by upregulating p21Cip1 and p27Kip1. Oncotarget.

[6] Dangle PP, Zaharieva B, Jia H, Pohar KS (2009). Ras-MAPK pathway as a therapeutic target in cancer - emphasis on bladder cancer. Recent Pat Anticancer Drug Discov.

[7] Dano K, Romer J, Nielsen BS, Bjorn S, Pyke C, Rygaard J (1999). Cancer invasion and tissue remodeling - cooperation of protease systems and cell types. APMIS.

[8] Davies B, Waxman J, Wasan H, Abel P, Williams G, Krausz T (1993). Levels of matrix metalloproteases in bladder cancer correlate with tumor grade and invasion. Cancer Res.

[9] Gerhardt D, Bertola G, Dietrich F, Figueiro F, Zanotto-Filho A, Moreira Fonseca JC (2014). Boldine induces cell cycle arrest and apoptosis in T24 human bladder cancer cell line via regulation of ERK, AKT, and GSK-3beta. Urol Oncol.

[10] Haddad RI, Weinstein LJ, Wieczorek TJ, Bhattacharya N, Raftopoulos H, Oster MW (2004). A phase II clinical and pharmacodynamic study of E7070 in patients with metastatic, recurrent, or refractory squamous cell carcinoma of the head and neck: modulation of retinoblastoma protein phosphorylation by a novel chloroindolyl sulfonamide cell cycle inhibitor. Clin Cancer Res.

[11] Izutani Y, Yogosawa S, Sowa Y, Sakai T (2012). Brassinin induces G1 phase arrest through increase of p21 and p27 by inhibition of the phosphatidylinositol 3-kinase signaling pathway in human colon cancer cells. Int J Oncol.

[12] Kamei K, Matsuoka H, Furuhata SI, Fujisaki RI, Kawakami T, Mogi S (2000). Anti-malarial activity of leaf-extract of hydrangea macrophylla, a common Japanese plant. Acta Med Okayama.

[13] Kim HJ, Kang CH, Jayasooriya R, Dilshara MG, Lee S, Choi YH (2016). Hydrangenol inhibits lipopolysaccharide-induced nitric oxide production in BV2 microglial cells by suppressing the NF-kappaB pathway and activating the Nrf2-mediated HO-1 pathway. Int Immunopharmacol.

[14] Lee MH, Yang HY (2001). Negative regulators of cyclin-dependent kinases and their roles in cancers. Cell Mol Life Sci.

[15] Li A, Blow JJ (2001). The origin of CDK regulation. Nat Cell Biol.

[16] Li MH, Ito D, Sanada M, Odani T, Hatori M, Iwase M (2004). Effect of 5-fluorouracil on G1 phase cell cycle regulation in oral cancer cell lines. Oral Oncol.

[17] Li T, Christensen SD, Frankel PH, Margolin KA, Agarwala SS, Luu T (2012). A phase II study of cell cycle inhibitor UCN-01 in patients with metastatic melanoma: a California Cancer Consortium trial. Invest New Drugs.

[18] Lin HL, Chang YF, Liu TY, Wu CW, Chi CW (1998). Submicromolar paclitaxel induces apoptosis in human gastric cancer cells at early G1 phase. Anticancer Res.

[19] Matsuda H, Shimoda H, Yamahara J, Yoshikawa M (1999). Effects of phyllodulcin, hydrangenol, and their 8-O-glucosides, and thunberginols A and F from Hydrangea macrophylla SERINGE var. thunbergii MAKINO on passive cutaneous anaphylaxis reaction in rats. Biol Pharm Bull.

[20] Michel L, Ley J, Wildes TM, Schaffer A, Robinson A, Chun SE (2016). Phase I trial of palbociclib, a selective cyclin dependent kinase 4/6 inhibitor, in combination with cetuximab in patients with recurrent/metastatic head and neck squamous cell carcinoma. Oral Oncol.

[21] Nozawa K, Yamada M, Tsuda Y, Kawai K, Nakajima S (1981). Antifungal activity of oosponol, oospolactone, phyllodulcin, hydrangenol, and some other related compounds. Chem Pharm Bull (Tokyo).

[22] Nutt JE, Durkan GC, Mellon JK, Lunec J (2003). Matrix metalloproteinases (MMPs) in bladder cancer: the induction of MMP9 by epidermal growth factor and its detection in urine. BJU Int.

[23] Osborne CK, Boldt DH, Clark GM, Trent JM (1983). Effects of tamoxifen on human breast cancer cell cycle kinetics: accumulation of cells in early G1 phase. Cancer Res.

[24] Owa T, Yoshino H, Yoshimatsu K, Nagasu T (2001). Cell cycle regulation in the G1 phase: a promising target for the development of new chemotherapeutic anticancer agents. Curr Med Chem.

[25] Park SL, Lee EJ, Kim WJ, Moon SK (2014). p27KIP1 is involved in ERK1/2-mediated MMP-9 expression via the activation of NF-kappaB binding in the IL-7-induced migration and invasion of 5637 cells. Int J Oncol.

[26] Sherr CJ (1996). Cancer cell cycles. Science.

[27] Siegel RL, Miller KD, Jemal A (2018). Cancer statistics, 2018. CA Cancer J Clin.

[28] Sugie S, Okamoto K, Rahman KM, Tanaka T, Kawai K, Yamahara J (1998). Inhibitory effects of plumbagin and juglone on azoxymethane-induced intestinal carcinogenesis in rats. Cancer Lett.

[29] Vadlakonda L, Pasupuleti M, Pallu R (2013). Role of PI3K-AKT-mTOR and Wnt signaling pathways in transition of G1-S phase of cell cycle in cancer cells. Front Oncol.

[30] Vasala K, Paakko P, Turpeenniemi-Hujanen T (2008). Matrix metalloproteinase-9 (MMP-9) immunoreactive protein in urinary bladder cancer: a marker of favorable prognosis. Anticancer Res.

[31] Wang L, Wang G, Yang D, Guo X, Xu Y, Feng B (2013). Euphol arrests breast cancer cells at the G1 phase through the modulation of cyclin D1, p21 and p27 expression. Mol Med Rep.

[32] Yuan L, Zhang Y, Xia J, Liu B, Zhang Q, Liu J (2015). Resveratrol induces cell cycle arrest via a p53-independent pathway in A549 cells. Mol Med Rep.

[33] Zhang H, Matsuda H, Kumahara A, Ito Y, Nakamura S, Yoshikawa M (2007). New type of anti-diabetic compounds from the processed leaves of Hydrangea macrophylla var. thunbergii (Hydrangeae Dulcis Folium). Bioorg Med Chem Lett.

[34] Zheng X, Ou Y, Shu M, Wang Y, Zhou Y, Su X (2014). Cholera toxin, a typical protein kinase A activator, induces G1 phase growth arrest in human bladder transitional cell carcinoma cells via inhibiting the c-Raf/MEK/ERK signaling pathway. Mol Med Rep.

